# Long-term exposure to estrogen enhances chemotherapeutic efficacy potentially through epigenetic mechanism in human breast cancer cells

**DOI:** 10.1371/journal.pone.0174227

**Published:** 2017-03-21

**Authors:** Yu-Wei Chang, Kamaleshwar P. Singh

**Affiliations:** Department of Environmental Toxicology, The Institute of Environmental and Human Health (TIEHH), Texas Tech University, Lubbock, Texas, United States of America; University of South Alabama Mitchell Cancer Institute, UNITED STATES

## Abstract

Chemotherapy is the most common clinical option for treatment of breast cancer. However, the efficacy of chemotherapy depends on the age of breast cancer patients. Breast tissues are estrogen responsive and the levels of ovarian estrogen vary among the breast cancer patients primarily between pre- and post-menopausal age. Whether this age-dependent variation in estrogen levels influences the chemotherapeutic efficacy in breast cancer patients is not known. Therefore, the objective of this study was to evaluate the effects of natural estrogen 17 beta-estradiol (E2) on the efficacy of chemotherapeutic drugs in breast cancer cells. Estrogen responsive MCF-7 and T47D breast cancer cells were long-term exposed to 100 pg/ml estrogen, and using these cells the efficacy of chemotherapeutic drugs doxorubicin and cisplatin were determined. The result of cell viability and cell cycle analysis revealed increased sensitivities of doxorubicin and cisplatin in estrogen-exposed MCF-7 and T47D cells as compared to their respective control cells. Gene expression analysis of cell cycle, anti-apoptosis, DNA repair, and drug transporter genes further confirmed the increased efficacy of chemotherapeutic drugs in estrogen-exposed cells at molecular level. To further understand the role of epigenetic mechanism in enhanced chemotherapeutic efficacy by estrogen, cells were pre-treated with epigenetic drugs, 5-aza-2-deoxycytidine and Trichostatin A prior to doxorubicin and cisplatin treatments. The 5-aza-2 deoxycytidine pre-treatment significantly decreased the estrogen-induced efficacy of doxorubicin and cisplatin, suggesting the role of estrogen-induced hypermethylation in enhanced sensitivity of these drugs in estrogen-exposed cells. In summary, the results of this study revealed that sensitivity to chemotherapy depends on the levels of estrogen in breast cancer cells. Findings of this study will have clinical implications in selecting the chemotherapy strategies for treatment of breast cancer patients depending on the serum estrogen levels that varies among pre- and post-menopausal age of the patients.

## Introduction

Breast cancer is a disease that includes multiple subtypes with different biological features, and response to clinical treatments also varies depending on the subtypes of this disease. Breast cancers are classified into subtypes based on several biological characteristics, such as, tumor size and grade, lymph node involvement, estrogen receptors (ER), progesterone receptors (PR) and gene expression profiling, such as human epidermal growth factor receptor 2 (EGFR2) expression [1, 2]. These biological characteristics of breast cancer itself are being used as targets in cancer treatment. For example, herceptin and lapatinib are used for HER2-positive breast cancer, whereas palbociclib and everolimus are used for ER-positive and HER2-negative breast cancer. Hormone therapy is another option because some types of breast cancer are affected by hormone in blood. For women with ER-positive breast cancer, tamoxifen is a drug designed to block estrogen receptors as an anti-estrogen [3–6].

Among the various therapeutic options, the chemotherapy is most common clinical option for treatment of breast cancer. Chemotherapy results in improved overall survival and significantly decreases the risk of recurrence and death in early-stage breast cancer patients [7, 8]. Chemotherapeutic drugs are also used as adjuvant chemotherapy after surgery, to kill any remaining cancer cells or as neoadjuvant chemotherapy before surgery mainly in metastatic breast cancer to evaluate the responses of the drug. Therefore, chemotherapy is an important and most commonly used option for the treatment of breast cancer [9].

There are multiple chemotherapeutic drugs that are used for breast cancer treatment, and mechanistic basis through which these drugs target cancer cells also differ for each class of drugs. Among the chemotherapeutic drugs, the DNA damage-dependent cytotoxic drugs, such as doxorubicin and cisplatin, are most commonly used for treatment of breast cancer. These drugs are considered as DNA damage-inducing drugs, which might increase reactive oxygen species, form DNA adducts, disrupt DNA repair system, and ultimately leads to DNA damage-dependent apoptosis/cell death [10, 11].

In the past two decades, several randomized trials have revealed that the efficacy of various chemotherapeutic drugs also varies among early-stage breast cancer patients [7, 12, 13]. There are several factors that can influence the outcome of chemotherapy or sensitivity of chemotherapeutic drugs, such as tumor size, tumor grade, hormone receptor status, and age of patients. For example, the efficacy of chemotherapy has been shown to vary depending on the age of breast cancer patients. In the report by the Early Breast Cancer Trialists' Collaborative Group, poly-chemotherapy with two or more chemotherapeutic improves 10-year survival of patients by about 27% for age group under 50, 14% for the ages between 51–69, and 8% for patients aged between 60–69 [13]. Similar observation was also reported by Cole et al. suggesting that chemotherapy contributed benefits in older women, but the extent of benefits were not as large as in younger women [8]. These studies suggest that chemotherapeutic responses are greater in younger patients than elder patients.

Breast tissue is estrogen responsive and serum estrogen level varies depending on the age of the patients. The concentration of estrogen in serum of pre-menopausal women ranges from 30 pg/ml to 450 pg/ml whereas in post-menopausal women it ranges from 0–40 pg/ml [14, 15]. Estrogen is an important hormone for normal tissue development and it contributes to many physiological functions, including in the development of organs and secondary sex characteristics, the regulation of menstrual cycle and the reproduction in female [16]. However, the chronic exposure to elevated levels of estrogen is a known risk factor for breast cancer. Estrogen has been shown to induce growth of cancer cells through multiple pathways including estrogen receptor-mediated pathways [17] and by inflicting genotoxic effects by increasing mutation rate [18]. Therefore, the ER modulators, such as tamoxifen are used to block the estrogen-induced growth signaling via ER as a clinical treatment of ER-positive breast cancers [19].

Although the chemotherapeutic responses are known to vary between pre-menopausal and post-menopausal age of breast cancer patients, the influence of the variable estrogen levels in chemotherapeutic response is not clear. As mentioned above, the serum estrogen level is an important factor to distinguish the pre- and post-menopausal age of patients. Whether age-dependent and physiologically relevant estrogen levels in serum influence the efficacy of DNA damaging chemotherapeutic drugs is not known. Therefore, the objective of this study was to determine the effect of estrogen on efficacy of well established and commonly used chemotherapeutic drugs doxorubicin and cisplatin in breast cancer cells. Additionally, the molecular mechanism through which estrogen can influence the chemosensitivity was also evaluated.

## Materials and methods

### Chemicals

5-aza-2-deoxycytidine (5-aza-2’-dC), 17 beta-estradiol (E2), cis-diamineplatinum (II) dichloride (cisplatin), Doxorubicin HCl, 3-(4, 5-dimethylthiazol-2-yl)-2, 5-diphenyltetrazolium bromide (MTT) were purchased from Sigma-Aldrich (St. Louis, MO). Trizol reagent was purchased from Invitrogen, Inc. RT-PCR kit was purchased from Bio-Rad, Inc.

### Cell culture and treatments

MCF-7 and T47D breast cancer cells were purchased from ATCC. MCF-7 cells were maintained in DMEM/F12 growth medium supplemented with 10% fetal bovine serum. Similarly, the T47D cells were maintained in RPMI growth medium supplemented with 10% fetal bovine serum. These cells were long-term treated with 100 pg/ml of 17 beta-estradiol (E2) in their respective growth medium supplemented with 1% FBS for period of four months. From here onward in this article, the parental MCF-7 and T47D cells are referred as MCF-7^P^ and T47D^P^, respectively. Similarly, the long-term E2 treated MCF-7 and T47D cells are referred as MCF-7^E^ and T47D^E^, respectively. The MCF-7^E^ and T47D^E^ cells were treated with chemotherapeutic drugs 300 nM of cisplatin either alone or in combination with epigenetic therapeutic drugs 5-aza-2’-dC and TSA. Similarly, MCF-7^P^ and MCF-7^E^ cells were treated with 10nM of doxorubicin. T47D cells are known to be inherently more resistant to doxorubicin than MCF-7 cells [20, 21]. Therefore, T47D^P^ and T47D^E^ cells were treated with a relatively higher concentration, 40 nM of doxorubicin. Cell growth analysis, and migration assay were performed after 72 h of treatment, whereas for gene expression analysis, RNA was isolated at 24 h after treatment.

### MTT assay for cell growth

For MTT assay, cells were seeded in 96 well plate in DMEM/F12 supplemented with 10% FBS medium, allowed for 24 hr for cells to attach and then exposed to chemotherapeutic (doxorubicin, cisplatin) and epigenetic therapeutic drugs (5-aza-2’-dC and TSA) in 1% FBS medium for 72 hr. MTT solution (1 mg/ml final concentration) was added to each well and incubated at 37°C for 3 h. To solubilize the formazan crystals formed by mitochondrial activity in viable cells, the cell culture media was completely removed, cells were given a wash with 1X PBS, and then 150 μl DMSO was added into each well. To facilitate the solubilization of formazan in DMSO, the plates were incubated with gentle shaking for another 5 minutes and then the color intensity of each well was measured by using microplate reader at 570nm and 630nm absorbance. Each treatment was performed triplicates and each experiment was repeated twice.

### Flow cytometry for cell cycle analysis

The effect of chemotherapeutics on cell cycle was measured by flow cytometry. Control and treatment group of cells were collected by trypsinization and fixed in 70% ethanol for 24 hr at 4°C. Just before loading the cells in flow cytometer for analysis, fixed cells were collected by centrifugation, washed in 1X PBS, and then stained with Guava cell cycle reagent for 30 minutes. After staining, samples were analyzed by Guava Easy-Cyte HT flow cytometer by counting 5000 events and data were analyzed by Guava Incyte software to calculate the percentage of cells in different stages of cell cycle.

### RNA isolation and quantitative real-time PCR

Total RNA was isolated from control and treatment group of cells by using Trizol reagent. PCR reactions for gene expression at transcript levels were performed by using 75 ng total RNA and SYBR green RT-PCR kit, and amplified by MyiQ2 real-time PCR detection system. The primers of genes of interest are listed in Table 1. The PCR amplification conditions used are as follows: 50°C for 15 mins for reverse transcription, 95°C for 5 minutes to inactivation of reverse transcriptase, followed by 40 cycles with each cycle containing step 1 of denaturation at 95°C for 10 seconds and step 2 of primer annealing and extension at 60°C for 30 seconds. Cycle threshold (Ct) value of each gene was normalized by the Ct value of housekeeping gene GADPH. The fold changes of gene expression was calculated by delta-delta Ct method [22].

**Table 1:** List of genes with their forward and reverse primer sequences used for the gene expression analysis by real- time quantitative PCR.

Gene	Forward primer sequence (5’-3’)	Reverse primer sequence (5’-3’)	Size(base pair)
***GAPDH***	GGTGGTCTCCTCTGACTTCAACA	GTTGCTGTAGCCAAATTCGTTGT	116
***CyclinD1***	AACTACCTGGACCGCTTCCT	CCACTTGAGCTTGTTCACCA	204
***Bcl2***	GGATGCCTTTGTGGAACTG	AGCCTGCAGCTTTGTTTCAT	231
***Bax***	TTTGCTTCAGGGTTTCATCC	CAGTTGAAGTTGCCGTCAGA	246
***TOP2A***	GGGTTCTTGAGCCCCTTCAC	CCAATGTAGGTGTCTGGGCG	185
***MLH1***	TGGGACGAAGAAAAGGAATG	GATCAGGCAGGTTAGCAAGC	250
***MYH***	CAGAGGCTTTGAAGGCTACC	TGCAGCATGACCTCTGAGAC	458
***RAR-β 2***	CCACACCTAGAGGATAAGCACT	GTGGTACCCTGATGATTTGTCC	326
***MBD4***	CAACTGCTCACCAACCAGGA	AAAAGGTGACCGAGGAGGTG	184
***MSH2***	GCCATTTTGGAGAAAGGACA	CTCACATGGCACAAAACACC	228
***PARP1***	GCCCTAAAGGCTCAGAACGA	AAGGCACTTGCTGCTTGTTG	112
***OGG1***	GACAAGAGCCAGGCTAGCAG	CTCTTGGAAGTGGGAGTCCA	126

### Wound healing migration assay

Wound healing migration assay was performed to observe the migration ability of E2-exposed MCF-7 cells. MCF-7^P^ and MCF-7^E^ cells were seeded in cell culture dishes using DMEM medium with 10% FBS. At 50% confluence, two parallel wound scratches were made by using sterile plastic micropipette tips (200 μl) in each dish, culture medium was replaced with fresh medium and then culture dishes were incubated under normal cell-culture conditions. The migration of cells in wound area was observed daily and photomicrographs were taken to document the migration of cells.

### Statistical analysis

Two-tailed paired *t* test was used to evaluate statistical significance of the changes in each treatment group. Level of significance (α) was set at 0.05 and differences with *p*<0.05 were considered as significant differences.

## Results

### Long-term exposure of estrogen increased the sensitivity to doxorubicin and cisplatin in MCF-7 and T47D cells

**Fig 1:**
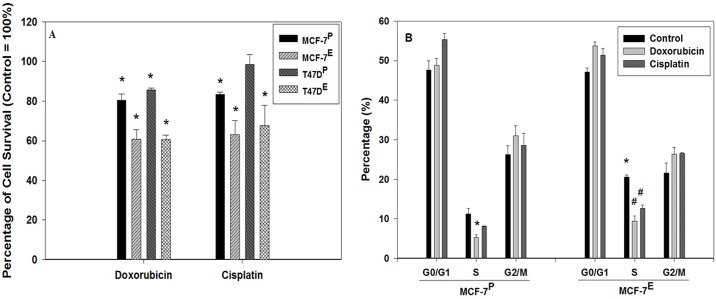
Enhanced sensitives to chemotherapeutic drugs doxorubicin and cisplatin in estrogen exposed MCF-7 and T47D cells as detected by MTT assay (A), and cell cycle analysis of estrogen exposed MCF-7 cells (B). (**A**) MTT assay was performed with doxorubicin and cisplatin treated cells as described in materials and methods section. MTT data was converted into percentage with control as 100% and histogram was plotted. Statistically significant change in the cell growth of doxorubicin and cisplatin treated groups as compared to their respective untreated control is indicated by an * symbol. (**B**) Histograms representing percentage of doxorubicin and cisplatin treated cells as well as their respective untreated control cells in G0/G1, S, and G2/M compartments of cell cycle. The error bars represent standard error of the mean (±SEM). Statistically significant (*p*<0.05) changes are indicated by the symbols * and #. The symbol * represents statistically significant (*p*<0.05) changes in S phase population from doxorubicin treated MCF-7^P^ (left panel) and untreated control MCF-7^E^ cells (right panel) as compared to the untreated control MCF-7^P^ cells. The symbol # indicates statistically significant changes in S phase population of MCF-7^E^ cells after doxorubicin and cisplatin treatments as compared to the untreated MCF-7^E^ cells.

To understand the effects of estrogen on sensitivity to chemotherapy, MCF-7^P^ and MCF-7^E^ cells were treated with 10 nM of doxorubicin or 300 nM of cisplatin for 72 hr. Considering the T47D cells being inherently more resistant to doxorubicin than MCF-7 cells, a relatively higher concentration, 40 nM of doxorubicin was used to treat T47D^P^ and T47D^E^ cells. The data of MTT assay revealed that doxorubicin and cisplatin treatment resulted in 39.9% and 37% decrease respectively in cell viability of MCF-7^E^ cells as compared to untreated MCF-7^E^ control cells. The same dose of doxorubicin and cisplatin caused just 19.6% and 16.5% decrease respectively in viability of MCF-7^P^ as compared to untreated MCF-7^P^ cells (Fig 1A). Similarly, doxorubicin and cisplatin treatments resulted in 39.4% and 32.3%, respectively, in decreased viability of T47D^E^ cells as compared to the untreated control T47D^E^ cells. The same doses of doxorubicin and cisplatin, however, caused a much less decrease by 14.3% and 1.5%, respectively, in the viability of T47D^P^ cells (Fig 1A). Therefore, the MTT data suggest that long-term E2 exposure increases the chemotherapeutic drugs sensitivity in MCF-7 and T47D breast cancer cells.

Cell cycle analysis by flow cytometry further confirmed the increased sensitivity of E2 exposed cells to doxorubicin and cisplatin. In MCF-7^P^ cells, the percentages of S phase population of cells were 11.3, 5.3, and 8.1% in control, doxorubicin and cisplatin treatment groups respectively. In MCF-7^E^ cells, the percentages of cells in S phase were 20.6, 9.4, and 12.7% in control, doxorubicin, and cisplatin treated groups respectively (Fig 1B). The comparison of S phase population revealed an increase by 9.3% in MCF-7^E^ cells from that of MCF-7^P^ cells suggesting that E2 increased the cell proliferation. Similarly, there was a decrease in S phase population of cells by 6.0% and 3.2% due to doxorubicin and cisplatin treatment in MCF-7^P^ cells. However, in MCF-7^E^ cells, a greater decrease in S phase population of cells by 11.2% and 7.9% due to doxorubicin and cisplatin treatment respectively was observed (Fig 1B).

**Fig 2:**
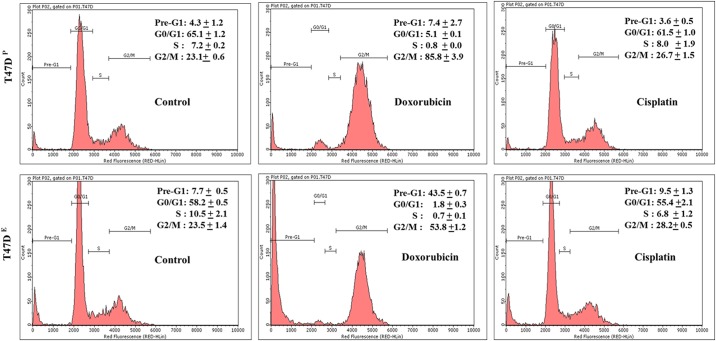
Flow cytometry analysis of cell cycle. Representative flow cytometry histograms of percentage of cells in Pre-G1 (apoptotic cells), G0/G1, S and G2/M phases of cell cycle from T47D^P^ cells (upper panel) and T47D^E^ cells (lower panel) cells treated with doxorubicin and cisplatin or untreated control for 72 hrs. Control and treated cells were fixed, stained, and analyzed by flow cytometry as described in materials and methods.

The cell cycle analysis of T47D^E^ cells revealed that increased sensitivity of chemotherapeutic drugs resulted not only in greater decrease of the S phase population of cells but also the percentage of pro-apoptotic pre-G1 population of cells (Fig 2). For example, the percentage of S phase decreased from 7.2% in untreated control T47D^P^ cells to 0.8% in doxorubicin treated T47D^P^ cells. However, the same dose of doxorubicin resulted a greater decrease from 10.5% in control T47D^E^ cells to 0.7% in doxorubicin treated T47D^E^ cells. There was no significant difference in S phase between cisplatin treated T47D^P^ and untreated control T47D^P^ cells. However, same dose of cisplatin resulted in decrease of S phase population from 10.5% in untreated control T47D^E^ cells to 6.8% in cisplatin treated T47D^E^ cells (Fig 2). The comparison of S phase population of 7.2% in untreated control T47D^P^ cells to that of 10.5% in control T47D^E^ cells also confirmed the effect of estrogen in increased proliferation in this cell line. Interestingly, the percentages of pro-apoptotic pre-G1 population of cells were also much higher in doxorubicin and cisplatin treated T47D^E^ cells as compared to the T47D^P^ cells (Fig 2). For example, the percentage of pre-G1 increased from 4.3% in untreated control T47D^P^ cells to 7.4% in doxorubicin treated T47D^P^ cells. However, the same dose of doxorubicin resulted a greater increase from 7.7% in untreated control T47D^E^ cells to 43.5% in doxorubicin treated T47D^E^ cells. Cisplatin treatment also resulted in a modest increase by 1.8% in pre-G1 population in T47D^E^ cells as compared to untreated control T47D^E^ cells. Therefore, the cell cycle data further confirmed the increased sensitivity of these chemotherapeutic drugs in MCF-7 and T47D breast cancer cells exposed to estrogen.

### Changes in expression of DNA repair and drug transporter genes in MCF-7^E^ cells

**Fig 3:**
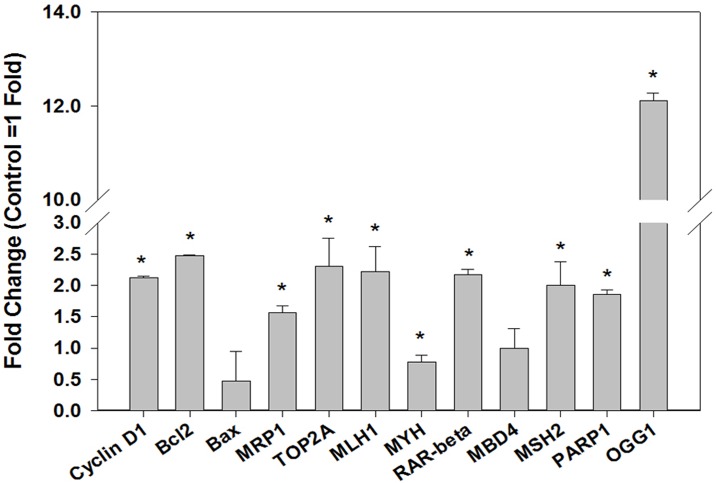
Real time quantitative reverse transcription PCR analysis of the expression of genes related to cell proliferation or survival, DNA repair, and drug transporters in MCF-7^E^ cells as compared to MCF-7^P^ cells. One-step real-time quantitative reverse transcription PCR for gene expression at transcript level was performed as described in materials and methods. The gene expression in fold-change was calculated by delta-delta Ct method. Histograms were plotted by using the average of triplicate values. The error bars represent standard error of the mean (±SEM). Statistically significant (*p*<0.05) changes are indicated by symbol *.

Altered expression of genes associated with DNA damage-dependent apoptotic response, and drug transporters influence chemotherapeutic response. Therefore, the expression of genes involved in oxidative DNA damage repair (OGG1, MYH), DNA repair-dependent apoptotic pathway (MLH1, MBD4, MSH2, PARP 1, RAR-β) and drug transports (MRP1, TOP2A) were measured to understand their role in increased sensitivity of doxorubicin and cisplatin in MCF-7^E^ cells. The results of quantitative real-time PCR analysis revealed 12.1, 2.2, 2.0, 1.9, and 2.2 folds increased expression of OGG1, MLH1, MSH2, PARP 1, and RAR-β respectively in MCF-7^E^ cells as compared to MCF-7^P^ cells (Fig 3). There was no change in MBD4 expression, but MYH gene expression was decreased by 1.25 folds in MCF-7^E^ cells. Similarly, the expression of drug transporters MRP1 and TOP 2A was increased by 1.6 and 2.3 folds respectively in MCF-7^E^ cells as compared to MCF-7^P^ cells (Fig 3). All these changes in expression of genes were statistically significant (*p* < 0.05).

### Epigenetic drugs diminished the estrogen-induced sensitivity to chemotherapy in MCF-7 cells

**Fig 4:**
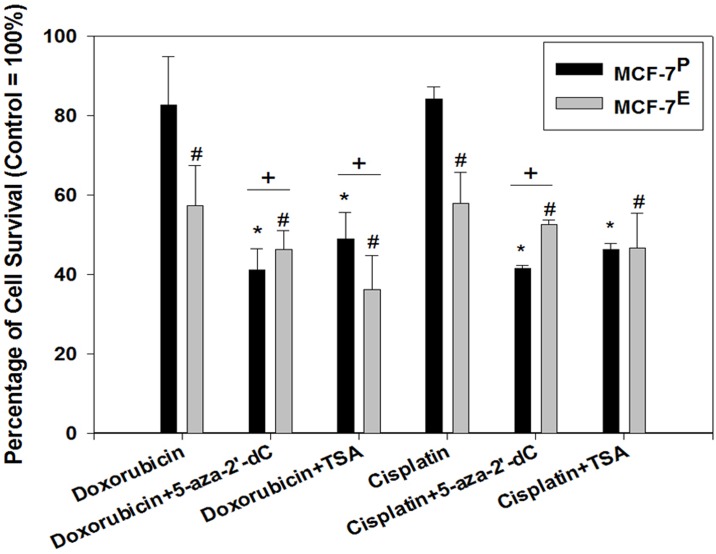
Effect of DNA demethylating agent 5 -aza-2’-dC and HDAC inhibitor TSA on sensitivity of chemotherapeutic drugs doxorubicin and cisplatin in MCF-7^P^ and MCF-7^E^ cells as determined by MTT assay for cell viability. Cells were first pre-treated with 5 -aza-2’-dC and TSA for 24 hr before doxorubicin and cisplatin treatments. The effect of 5 -aza-2’-dC and TSA pre-treatments on chemotherapeutic sensitivity was then determined by MTT assay as described in materials and methods. MTT data was converted into percentage with their respective controls as 100% and histogram was plotted. The error bars represent standard error of the mean (±SEM). Symbols * represent statistically significant difference in viability of various treatment groups (5 -aza-2’-dC+ Doxorubicin, TSA+ Doxorubicin, 5 -aza-2’-dC+ Cisplatin, TSA+ Cisplatin) of MCF-7^P^ as compared to its untreated MCF-7^P^ control cells. The symbol # represents statistically significant difference in viability of various treatment groups (Doxorubicin, 5 -aza-2’-dC+ Doxorubicin, TSA+ Doxorubicin, Cisplatin, 5 -aza-2’-dC+ Cisplatin, TSA+ Cisplatin) of MCF-7^E^ cells as compared to its untreated MCF-7^E^ control cells. The symbol + represents statistically significant difference in cell viability between MCF-7^E^ cells and MCF-7^P^ cells within each treatment groups (5 -aza-2’-dC+ Doxorubicin, TSA+ Doxorubicin, 5 -aza-2’-dC+ Cisplatin).

To further understand the role of E2-induced epigenetic alterations in increased sensitivity to chemotherapeutic drugs, these cells were first pre-treated with the epigenetic therapeutics, such as, DNA demethylating agent 5-aza-2-deoxycytidine (5-aza-2’-dC) or histone deacetylase inhibitor Trichostatin A (TSA) and then exposed to doxorubicin or cisplatin. After initial optimization of doses, the 2μM of 5-aza-2’-dC and 25 nM of TSA were found to be non-cytotoxic and therefore were selected for pre-treatment of MCF-7 cells in this study. The viability of MCF-7^E^ and MCF-7^P^ cells after doxorubicin and cisplatin treatments were calculated as percentage relative to their respective untreated (without doxorubicin and cisplatin) control cells. After doxorubicin treatment, the percentage of cell viability decreased from 82.7% in MCF-7^P^ cells to 57.4% in MCF-7^E^ cells, suggesting that E2 exposure resulted in 25.3% increase in sensitivity to doxorubicin (Fig 4). However, in 5-aza-2’-dC pretreated group of cells, after doxorubicin treatment the percentage of cell viability increased from 41.2% in MCF-7^P^ cells to 46.3% in MCF-7^E^ cells. Similarly, after cisplatin treatment the percentage of cell viability decreased from 84.2% in MCF-7^P^ cells to 58.0% in MCF-7^E^ cells, suggesting that E2 exposure resulted in 26.2% increase in sensitivity to cisplatin (Fig 4). Contrary to this, in 5-aza-2’-dC pretreated group of cells, after cisplatin treatment the percentage of cell viability increased from 41.6% in MCF-7^P^ cells to 52.5% in MCF-7^E^ cells. This 5.1% and 10.9% decreased sensitivity to doxorubicin and cisplatin respectively in MCF-7^E^ cells as compared to MCF-7^P^ cells after 5-aza-2’-dC pre-treatment suggest that E2-induced DNA hypermethylation could potentially be involved in increased sensitivity to these chemotherapeutic drugs in MCF-7^E^ cells. TSA did not change the increased doxorubicin sensitivity in MCF-7^E^ cells. However, the increased sensitivity to cisplatin in MCF-7^E^ cells was partially diminished by TSA pre-treatment (Fig 4).

### Increased migration potential of MCF-7^E^ cells was reversed by epigenetic therapeutics

**Fig 5:**
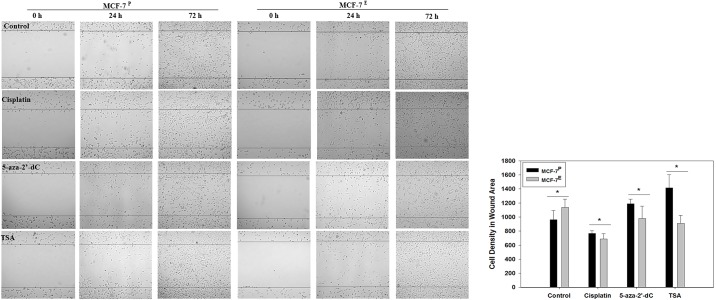
Representative photomicrographs (40 X) of wound healing assay (left panel) and histograms of density of cells migrated into wounded area (right panel) from MCF-7^E^ and MCF-7^P^ cells treated with cisplatin, 5 -aza-2’-dC, TSA and their respective untreated control cells. In left panel, cells were seeded in dishes and wound healing assay was performed as described in materials and methods. Migration of cells into wounded area was observed under microscope at 0, 24, and 72 hrs. Density of cells migrated into wounded area at 72 hr was quantified by ImageJ software and the histogram was plotted using the data of cell density in the right panel. The cell density data is given in arbitrary units. The error bars represent standard error of the mean (±SEM). Statistically significant (*p*<0.05) changes between MCF-7^E^ and MCF-7^P^ cells within each treatment groups are indicated by symbol *.

In order to observe the effects of estrogen on cell migration potential, wound healing migration assay was performed. The migration of cells in wound area from both the MCF-7^E^ and MCF-7^P^ cells was observed at 24 and 72 hrs. Photomicrographs of wound healing are given in Fig 5, left panel, and quantified data of cell density in the wound area as determined by ImageJ software is presented as histogram in Fig 5, right panel. The number of cells migrated in the wound area were observed to be more in MCF-7^E^ cells than that of the MCF-7^P^ cells (Fig 5). The treatment of MCF-7^E^ cells with cisplatin, 5-aza-2’-dC and TSA resulted in less migration of cells as compared to untreated MCF-7^E^ cells, suggesting that increased migration of MCF-7^E^ cells could potentially be due to E2-induced epigenetic alterations. The analysis of cell numbers in the wound area further confirmed the statistically significant increase in migration of MCF-7^E^ cells and this increase was reduced by 5-aza-2’-dC and TSA treatments (Fig 5).

## Discussion

The most important and novel finding of this study is that the pre-treatment with estrogen significantly enhances the cytotoxic effects of doxorubicin and cisplatin in estrogen responsive breast cancer cells. Cytotoxic chemotherapy is a well-established treatment option for all subtypes of breast cancers. Previous studies have shown that the effectiveness of cytotoxic chemotherapy decreases with the increased age of breast cancer patients [12, 13]. Although, several factors, such as, reduction of body function and multiple comorbidities have been implicated in increased age-associated decreased efficacy of cytotoxic chemotherapy [8]. However, the effects of estrogen level, that significantly varies depending on the pre- and post-menopausal age of the breast cancer patients, on sensitivity to chemotherapy are not clear [23, 24]. The findings of this study suggest that pre-treatment with estrogen followed by chemotherapy would significantly enhance the efficacy of the later in breast cancer cells. The increased cell proliferation by estrogen pretreatment that creates a suitable cellular microenvironment for accumulation of DNA damage and consequently DNA damaged-induced apoptosis by cytotoxic chemotherapeutic drugs doxorubicin and cisplatin is another important finding of this study. Therefore, to our knowledge, this is the first report that revealed not only the enhanced efficacy of DNA damaging chemotherapeutic drugs doxorubicin and cisplatin, but also suggested an epigenetic basis for this enhanced efficacy by estrogen pretreatment followed by chemotherapy in breast cancer cells.

Previous studies have compared the efficacy of endocrine therapy with that of cytotoxic chemotherapy. Endocrine therapy using estrogen antagonist, such as tamoxifen, followed by cytotoxic chemotherapy has been shown to give better result in terms of breast cancer treatment [25, 26]. The principle behind this strategy is that estrogen antagonist tamoxifen blocks the estrogen receptor and therefore inhibition of estrogen-induced growth of breast cancer cells followed by chemotherapy that induces cytotoxicity.

Estrogen is known to induce cell proliferation in breast cancer cells [27]. Highly proliferative cells with actively replicating DNA are more prone to DNA damage than less proliferative cells [28]. In fact, this is the principle on which chemotherapy targets actively dividing cancer cells than the normal cells [29, 30]. The data of MTT assay, cell cycle analysis, and expression of Cyclin D1, Bcl2, and Bax revealed that estrogen treatment induced proliferation in MCF-7 cells. Therefore, logical explanation for mechanism behind the increased efficacy of doxorubicin and cisplatin in estrogen exposed MCF-7 and T47D cells would be that these cells with actively replicating DNA are targeted more by these DNA damaging chemotherapeutic drugs and ultimately more prone to severe DNA damage-induced apoptosis.

Drug transporters, such as multidrug resistance associated protein 1 (MRP1), are known to influence the sensitivity of cancer cells to chemotherapy [31, 32]. In the present study, the expression level of drug transporters MRP1 was increased in MCF-7^E^ cells. MRP1 is an efflux transporter that takes the toxic chemicals or metabolites out of cells to protect them from the toxicity of xenobiotics. Previous studies have shown the altered expression of MRP1 associated with the drug resistance in cancer cells [32, 33]. In this study, however, an increase in MRP1 expression was observed and yet the cells were more sensitive to doxorubicin and cisplatin. The reasonable explanation for this increase in MRP1 expression could be a feedback response to adapt to the increase cytotoxicity by doxorubicin. Additionally, the reports also suggest that MRP1 export the metabolite of estrogen out of cells [34–36]. Therefore, the other explanation for the observed increase in MRP1 expression in E2-treated cells could be for the purpose to efflux of E2 metabolite out of cells. Breast cancer cells are known to have high levels of β-glucuronidase that can convert estradiol to estrone glucuronide, which can then be transported out from the cells by MRP1 [36, 37].

Topoisomerase IIa (TOP 2A) is an important target for doxorubicin to induce cytotoxicity [38]. TOP2A is ATP-dependent enzyme and plays a role in releasing twisted double strands of replicating DNA [39, 40]. The expression of TOP2A is at highest level during G2/M phase, but it is also expressed during S phase [41–43]. The alteration in TOP2A expression has been reported to influence the efficacy of chemotherapy [44]. The expression level of TOP2A is a key indicator for the efficacy of doxorubicin, and both in vivo and in vitro studies have shown that suppressed TOP2A expression leads to increase resistance against doxorubicin [45]. Additional evidence suggests that high levels of TOP2A expression is associated with increased sensitivity to doxorubicin in cervix and breast cancer cells [38, 46]. In this study, the increased expression of TOP2A was observed in MCF-7^E^ cells. Therefore, our data of increased TOP2A expression and increased sensitivity to doxorubicin in E2 exposed cells further supports the view that higher levels of TOP2A serve as target for doxorubicin and facilitates the increased cytotoxic effects of doxorubicin in cancer cells.

Estrogen has been reported as genotoxic agents causing oxidative DNA damage, DNA adducts and gene mutations in breast cancer cells [47]. Estrogen-induced cell proliferation can promote DNA damage accumulation because of insufficient timely repair [48]. DNA damages are repaired by multiple mechanism such as base excision repair (BER), nucleotide excision repair (NER) and mismatch repair (MMR) [49]. Additionally, the MMR system has been known to play an important role in cellular response to DNA damage, cell cycle arrest, apoptosis [50–52] and also to the damage caused by cisplatin [53, 54]. MLH1 is well known to play important roles in MMR-mediated apoptotic response [55, 56]. Similarly, the mismatch repair protein MSH2 also mediates DNA damage–induced apoptosis [51, 57]. The results of this study revealed the increased expression of MMR-related genes MLH1 and MSH2 in MCF-7^E^ cells. These previous reports on the role of MMR genes in apoptosis and the findings of this study showing their increased expression in MCF-7^E^ cells that were more sensitive to doxorubicin and cisplatin suggest that MMR genes act as mediators for the chemotherapeutic drugs-induced cytotoxicity.

In addition to MMR genes, the increase in the expression of OGG1 and PARP 1 were also observed in this study. OGG1 is a DNA repair gene involved in BER pathway and is also a known marker for oxidative DNA damage [58]. Similarly, PARP 1 is a known regulator of E2-induced cell growth [59, 60]. E2 has been shown to induce oxidative DNA damage [61], and is also known to induce cell proliferation [62]. Therefore, the increased expression of OGG1 and PARP1 in MCF-7^E^ cells not only further confirmed the E2-induced proliferation but also indicated the E2-induced oxidative DNA damage in these cells. Both, the increased number of cells in replicating S phase and accumulation of oxidative DNA damage by long-term E2 exposure could potentially further increase the sensitivity of cytotoxic and DNA damaging chemotherapeutic drugs such as doxorubicin and cisplatin.

In our data, epigenetic drugs decreased the sensitivity to doxorubicin and cisplatin in MCF-7^E^ cells, and inhibited migration ability that was induced by estrogen. Epigenetic modifications can cause the changes in the expression of various classes of genes such as DNA repair system, GSH-related detoxification, and influx/efflux transporters in cells [63]. Estrogen has also been shown to cause changes in the expression of epigenetic regulatory genes and epigenetic modifications [64–66]. The exact mechanism through which these epigenetic-based therapeutics antagonizes the doxorubicin and cisplatin sensitivity in E2-exposed cells is not clear and needs further investigation. However, based on previous reports of estrogen-induced epigenetic changes, it is logical to guess that the antagonistic effects of epigenetic therapeutics on the sensitivity of doxorubicin and cisplatin could be due to reversal of estrogen-induced epigenetic changes that made these cells more sensitive to these chemotherapeutic drugs.

In summary, findings of this study suggest that estrogen-pretreatment followed by chemotherapy would be a better approach to enhance the efficacy of DNA damaging cytotoxic drugs in estrogen receptor-positive breast cancer cells. This study will have significant clinical implications in selecting the type of chemotherapy in breast cancer patients at pre- and post-menopausal age, and potential of estrogen-pretreatment followed by chemotherapy in post-menopausal breast cancer patients with estrogen receptor-positive tumors.

## Supporting information

S1 File Raw data file for MTT assay from MCF-7 cells that were used for Fig 1A. MTT assay was performed as described in materials and methods. The data from plate reader was exported to excel file for analysis. The absorbance value at 570 nm was substracted from absorbance value at 630 nm (background) for each well, average of the triplicate values were calculated, and then values were converted to percentage of control with control as 100%. Using these values, the graph were plotted as given in Fig 1A.
(PDF)

S2 File Raw data file for cell cycle analysis of MCF-7 cells that were used to plot Fig 1B. The cell cycle analysis was performed as described in material and methods. The data from flow cytometer was exported to excel file for further analysis. The average of the replicates with the standard error of the mean (±SEM) were calculated and using these values the graph were plotted as given in Fig 1B.
(PDF)
