# Supplementary material for: Long-term exposure to estrogen enhances chemotherapeutic efficacy potentially through epigenetic mechanism in human breast cancer cells
Source: PLoS One. 2017 Mar 21;12(3):e0174227. doi: 10.1371/journal.pone.0174227 (PMC5360320; doi:10.1371/journal.pone.0174227)
Supplement: S1 File — MTT assay was performed as described in materials and methods. The data from plate reader was exported to excel file for analysis. The absorbance value at 570 nm was substracted from absorbance value at 630 nm (background) for each well, average of the triplicate values were calculated, and then values were converted to percentage of control with control as 100%. Using these values, the graph were plotted as given in Fig 1A. (PDF) [file pone.0174227.s001.pdf]

Raw data for absorbance value from plate reader

570nm

1	2	3	4	5
0.43	0.4788	0.49	0.4562	0.4408
0.4934	0.3982	0.5428	0.4069	0.4484
0.5319	0.4069	0.3943	0.4394	0.4337
0.4972	0.4214	0.5558	0.5295	0.5728
0.4393	0.4591	0.427	0.5521	0.5286
0.4846	0.5171	0.554	0.4671	0.5302
0.4108	0.3699	0.4751	0.4917	0.4868
0.4474	0.4431	0.4492	0.5025	0.5214

630nm

1	2	3	4	5
0.1691	0.1858	0.1871	0.176	0.1713
0.1895	0.1591	0.2037	0.159	0.1723
0.2003	0.1598	0.1554	0.1694	0.1708
0.1977	0.1648	0.2061	0.2098	0.2162
0.1693	0.1756	0.1697	0.2193	0.2108
0.1841	0.2051	0.2071	0.1787	0.1993
0.1609	0.1493	0.1869	0.1882	0.1885
0.1723	0.171	0.177	0.1906	0.2014

570nm-630nm

	Untreated Control		
MCF-7P	0.2609	0.293	0.3029

MCF-7E

Untreated Control		
0.3005	0.312	0.3469

These final values were used and exported to sigmaplot to draw final figure 1A

					mean	mean/
	Untreated Control	0.2609	0.293	0.3029	0.2856	91.351541
MCF-7P	Cisplatin-300nM	0.2365	0.2379	0.2414	0.2386	99.119866
	Doxorubicin-10 nM	0.228	0.2335	0.2269	0.2294667	99.360837
	Untreated Control	0.3005	0.312	0.3469	0.3198	93.964978
MCF-7E	Cisplatin-300nM	0.2175	0.1921	0.1942	0.2012667	108.06558
	Doxorubicin-10 nM	0.2015	0.1825	0.1919	0.1919667	104.96614

6	7	8	9	10	11	12
0.4397	0.3807	0.3879	0.3788	0.2872	0.2446	0.2103
0.4659	0.4372	0.3992	0.4438	0.395	0.3978	0.4003
0.4012	0.3178	0.3861	0.3465	0.223	0.2606	0.2363
0.4254	0.4684	0.4452	0.4645	0.3877	0.3913	0.3928
0.4974	0.3514	0.3249	0.3627	0.2538	0.253	0.3501
0.6115	0.4346	0.4166	0.4813	0.3626	0.335	0.3411
0.455	0.4354	0.3085	0.3493	0.2876	0.2919	0.2022
0.5328	0.455	0.4988	0.4818	0.3434	0.433	0.516

6	7	8	9	10	11	12
0.1707	0.1527	0.1544	0.1519	0.1222	0.1083	0.0967
0.1791	0.1732	0.1652	0.1749	0.1585	0.1599	0.1589
0.1566	0.132	0.1541	0.1428	0.1002	0.1127	0.1047
0.1736	0.1821	0.1756	0.1892	0.1589	0.1559	0.1557
0.1963	0.1499	0.1424	0.1708	0.1106	0.109	0.1477
0.2263	0.172	0.1663	0.1888	0.1451	0.1429	0.1469
0.1797	0.1675	0.1342	0.1443	0.1236	0.1257	0.0936
0.2207	0.1747	0.2026	0.1851	0.1406	0.1697	0.2001

	Doxorubicin 10 nM				
0.228	0.2335	0.2269			
				Cisplatin 300nM	
			0.2365	0.2379	0.2414

	Doxorubicin 10 nM				
0.2015	0.1825	0.1919			
				Cisplatin 300nM	
			0.2175	0.1921	0.1942

'control mean*100		Normolizat	SEM	p-value
102.59104	106.05742	100	7.687707475	
99.706622	101.17351	83.543417	1.057789561	0.0211
101.7577	98.881464	80.345472	1.540966856	0.0120
97.560976	108.47405	100	7.555785515	
95.445512	96.488904	62.935168	7.004456311	0.0018
95.068588	99.965272	60.0271	4.948867218	0.0010
