# Supplementary material for: Long-term exposure to estrogen enhances chemotherapeutic efficacy potentially through epigenetic mechanism in human breast cancer cells
Source: PLoS One. 2017 Mar 21;12(3):e0174227. doi: 10.1371/journal.pone.0174227 (PMC5360320; doi:10.1371/journal.pone.0174227)
Supplement: S2 File — The cell cycle analysis was performed as described in material and methods. The data from flow cytometer was exported to excel file for further analysis. The average of the replicates with the standard error of the mean (±SEM) were calculated and using these values the graph were plotted as given in Fig 1B. (PDF) [file pone.0174227.s002.pdf]

raw data from flow cytometry

MCF-7P	G0/G1		S	
Untreated Control	46.0499	49.3089	10.34	12.2462
Cisplatin	56.3945	54.2313	8.16	8.00745
Doxorubicin	47.626	50.042	4.84	5.74761
MCF-7E				
Control	46.4286	47.8748	20.26	20.9558
Cisplatin	52.5765	50.2536	12.1	13.2278
Doxorubicin	52.9897	54.4252	8.47594	10.3764

Average of the replicates with standrad error od the mean and p-value

		G0/G1			S		
MCF-7P		Mean	SEM	p-value	Mean	SEM	p-value
	Untreated (	47.6794	2.304461		11.2931	1.3478869	
	Cisplatin	55.3129	1.5296134	0.0598153	8.083725	0.1078691	0.0784551
	Doxorubicir	48.834	1.70837	0.626618	5.293805	0.6417772	0.0295936
MCF-7E							
	Untreated (	47.1517	1.0226178	0.7951318	20.6079	0.4920049	0.0116575
	Cisplatin	51.41505	1.6425383	0.0893901	12.6639	0.797475	0.0068849
	Doxorubicir	53.70745	1.0150518	0.0233116	9.42617	1.3438282	0.0080905

These values were exported to sigmaplot for final graph as given figure 1B

G2/M

	24.64	27.7963
	30.7346	26.3397
	32.7988	29.256
	23.4	19.7716
	26.6707	26.3745
	27.582	25.0458

G2/M

Mean	SEM	p-value
26.21815	2.2318411	
28.53715	3.1076636	0.4816689
31.0274	2.5051379	0.1798594
21.5858	2.5656662	0.9372129
26.5226	0.209445	0.8162789
26.3139	1.7933642	0.8468075
